# Characteristics and influencing factors of Airbnb spatial distribution in China’s rapid urbanization process: A case study of Nanjing

**DOI:** 10.1371/journal.pone.0248647

**Published:** 2021-03-18

**Authors:** Shijie Sun, Shengyue Zhang, Xingjian Wang

**Affiliations:** 1 School of Architecture, Southeast University, Nanjing, Jiangsu, China; 2 Jiangsu Institute of Urban Planning and Design, Nanjing, Jiangsu, China; Northeastern University (Shenyang China), CHINA

## Abstract

As in other countries, short-term rentals for tourism services are growing rapidly in China’s tourist cities, which are mainly operated through Airbnb. This paper explores whether the spatial distribution of Airbnb in China’s rapid urbanization process exhibits characteristics, paths, and drivers that are different from those of cities in other countries. Airbnb is a model for the global sharing economy, but it is increasingly influenced by other functions and facilities in cities as it grows. In this paper, the zero-expansion negative binomial regression was used to study the factors affecting the spatial distribution of Airbnb in Nanjing, China. The results showed that the spatial distribution of Airbnb listings was correlated with the distribution of cultural attractions, universities, public transport accessibility, shopping centers, and business apartments. By analyzing the driving forces of Airbnb’s development in Nanjing, this paper found that a large number of business apartments developed in cities were essential providers of Airbnb listings, and affected its spatial distribution. The gap between short-term and long-term rentals was also correlated with the distribution of Airbnb. In addition, similar to the previous literature findings, the increase in the proportion of professional hosts changes the original intention of Airbnb for sharing and communication. Our empirical results applies to the current situation of Airbnb in Chinese cities, which is conducive to the government’s more intelligent management and effective promotion of the Airbnb market. Our findings also provide positive references for urban renewal policies and public participation methods in China.

## Introduction

The attractive concept of sharing accommodation represented by Airbnb has raised the attention of scholars. Founded in 2008, Airbnb has covered 65,000 cities worldwide by 2017, becoming the largest accommodation sharing platform in the world. Researchers analyzed the impact of Airbnb on the traditional hotel business [1–3] and housing [4–6]. Some studies believe that the rapid growth of Airbnb may bring community problems and promote gentrification of tourism [4, 7–9]. Therefore they also discuss the management of Airbnb [7, 10]. In recent years, researchers have studied the spatial distribution characteristics of some cities considering the potential economic, social, and cultural impacts that Airbnb might have on communities. These studies showed that the distribution of Airbnb listing was related to city centers, major attractions, and transport accessibility [11, 12].

In China, the construction of transportation infrastructure over the last two decades has greatly contributed to the tourism boom [13, 14], and the growth of shared urban accommodation has followed. According to the “Report on The Development of Shared Accommodation in China (2018)”, the transaction volume of shared accommodation in 2017 was around 14.5 billion yuan, which increased 70.6 percent compared to the previous year, and the number of domestic listings on major shared accommodation platforms was about 3 million. Airbnb officially entered the Chinese market in 2015 and expanded rapidly. In 2019, the web visits of Airbnb exceeded that of other accommodation-sharing platforms in China. In Nanjing, the selected site of this study, Airbnb has more short-term rental listings than its competitors. However, few studies discussed the correlation between it and urban elements. In particular, although Airbnb in China develops rapidly, there are gaps in the research of spatial distribution characteristics, and there is a lack of discussion on the spatial growth mechanism behind it. The above problems resulted in scholars have an insufficient understanding of the spatial growth characteristics of Airbnb in China.

Therefore, Airbnb’s spatial distribution characteristics and growth mechanism need to be better understood to deal with various related urban issues. This paper analyzes the spatial distribution characteristics of Airbnb in the central regions of Nanjing and tries to find the correlation between the distribution of listing and other characters of the city. The goal is to answer the following questions: What are the characteristics of the spatial distribution of Airbnb in Nanjing city? What factors determine the distribution and expansion of Airbnb? Answering these questions will help urban planners and policymakers deal with the problems caused by the proliferation of Airbnb in the urban renewal process.

## Literature review

### The influences of Airbnb

Some studies showed that the rapid growth of Airbnb might bring negative impacts on communities, including the increasing housing rental costs, social conflicts, security problems, and noise [6, 7, 15]. The most important one is that the rapid increase of Airbnb listings may lead to the rise in housing costs [16, 17]. A study of Boston found that the rental price increases by 0.4% when Airbnb listings increase one standard deviation [6]. According to Gurran and Phibbs (2017) ’s study of Sydney, it is found that Airbnb may cause community problems such as noise, congestion, and reduction of long-term rentable housing [7]. Cocola-gant and Gago (2019) tracked the Alfma community in Lisbon and found that the gentrification process experienced by the residents was an unfair social process [18]. In addition, it has been shown in many studies that the negative impact of Airbnb is geographically unbalanced [19]. The study of Gutierrez et al. (2017) about the spatial distribution of shared home-stays and hotels in Barcelona illustrated that home-stays are mainly clustered in the areas of city centers or famous tourist attractions, posing new challenges to the harmonious coexistence of local communities [20].

### Airbnb and rental gap

There are two main reasons for the negative impact of Airbnb. First, many Airbnb listings are located in residential areas where tourism infrastructure and carrying capacity are limited and insufficient. Therefore, Airbnb listings may cause tourism-oriented pressure in residential areas [7]. Furthermore, although the original intention of Airbnb is to make use of unused space, some houses are only used as Airbnb houses, most of which are downtown apartments rather than real empty ones, which may reduce the supply of long-rented listing [4, 5, 8]. Many researchers are in agreement that the rent gap between the long-term and short-term rental was the mainspring of convert leasing into an Airbnb. According to a study of New York City, Wachsmuth and Weisler (2018) cited and reconstructed the model of rent gap raised by Neil Smith. They claimed that the potential rental was raised by Airbnb listing, which widened the rent gap and eventually led to gentrification [4]. Smith’s classical rent gap theory holds that with the depreciation of buildings, the "actual rental" will gradually decline. In contrast, the "potential rental" will keep increasing, which forms the ever-expanding "rent difference" between the two. When the difference expands to meet the revenue expectation of capital, the structural incentive of capital reinvestment begins to appear, and it is easy to get gentrified [21]. Nonetheless, the rent gap brought by Airbnb may not accompany the redevelopment of cities, but rapidly increase the potential rental through short-term renting. Yrigoy (2018) also believed that the rent gap between short-term and long-term rental drive the landlord to convert the long-term rentals into the short-term ones, which resulted in the drop in the stock of rental housing in tourist cities and tourism gentrification [8]. Similarly, such a rent gap has become the main driving force of Airbnb’s expansion in China.

### Professional hosts of Airbnb

Professional hosts are the most controversial topic in the study of Airbnb. Numbers of international studies believe the professional hosts operating two or more Airbnb units are the main beneficiaries of Airbnb and may lead to a decrease in the number of long-term rental listings [6, 22–24]. According to Horn and Merante’s study, a host that operated more than two Airbnb apartments was defined as a professional host [6]. Murry, from Inside Airbnb, also defined a landlord who owned two or more houses as a professional host. Wachsmuth et al. (2017) defined the “triple threat” to Airbnb, including full-time listing, entire house properties, and multi-listing hosts (professional hosts) [23]. He defined a professional host more strictly, thinking that only a landlord with two or more full-house listings or three and more single-room listings could be considered as a professional host. He conducted researches on three cities in Canada and found that Airbnb listings cut down the number of long-term rental housing in the cities, and professional hosts were the main threat. Professional host is also a trending topic in China. According to the investigation of "2019 Urban Home Stay Entrepreneur Data Report", the average operating time of housing supply was about 0.5 hours, while the average time spent of 20 to 30 housing units would drop to about 0.1 hours per unit. Marginal costs were significantly lower, but revenues were higher, so hosts would expand or join escrow companies. In China, professional hosts and hosting companies are the mainstays of Airbnb’s operation. Professional hosts have led to disputes about pushing up rents and whether to reduce the sharing attributes of Airbnb.

### Spatial distribution of Airbnb

Studies on the spatial distribution characteristics of Airbnb mainly focued on the relationship between the distribution of Airbnb listings and the other factors, such as built environment, functional structure, and demographic characteristics of urban space [25–28]. Gutierrez et al. (2017) found a close spatial relationship between Airbnb and hotels through a case analysis of Barcelona, and it showed a clear central-peripheral pattern. Airbnb listings were mainly concentrated in the central area, which was related to the number of leisure and restaurants nearby, covering a wider area than the main axis of the hotel [20]. Comparing with the hotel industry, Airbnb took advantage of major tourist attractions in nearby cities and extended to residential areas in the city center, bringing tourism pressure into residential areas. Quattrone et al. (2016) analyzed the distribution of Airbnb apartments in London from 2012 to 2015. They assumed that a specific group would profit from Airbnb. The analysis showed that the homeownership rate was negatively correlated with the number of Airbnb apartments. The finding suggested that landlords make money from Airbnb by renting their houses rather than owning them [25]. In the analysis of the spatial distribution of Airbnb in Seoul, South Korea, Ki and Lee (2019) used the negative binaries regression model to test the factors influencing Airbnb’s location characteristics. The study showed that Airbnb’s units preferred to be located in areas near universities or subway stations, as well as areas with a high proportion of single families [12]. Xu et al. (2017), used the least-squares method and geographically weighted regression to study the distributive characteristics of Airbnb in London. They believed that the shared accommodation was mainly concentrated in the city center and the surrounding area of tourist attractions. Furthermore, the surrounding environmental factors, such as water, vegetation, universities, arts & human landscape, transport, and nightlife spots, were the significant factors that were affecting the spatial distribution of Airbnb [11]. In general, the above studies all agreed that the distribution of Airbnb listing was correlated with city centers, important scenic spots, and urban environmental elements.

This study used the research methods of Ki and Lee (2019) and Xu et al. (2017) [11, 12] to study the spatial distribution of Airbnb in Nanjing, but the major contribution of this work was to further highlight the coupling relationship with the urban spatial structure. At the same time, it studied the driving factors of Airbnb’s development from the aspects of urban development and housing source, professional hosts, and rent gap.

## Data and methods

### Study area

This study chose Nanjing as an example. Nanjing is a famous tourist city in China, the capital of Jiangsu Province, and one of the important central cities in east China, with a total population of about 8.5 million. Over the past three years, the number of Airbnb listings in Nanjing has increased nearly tenfold. It has been repeatedly reported by the news that there are various community problems caused by Airbnb, such as noise, fire risk, and safety problems.

The central region of Nanjing was selected as the research area, with a total area of about 295 km². The primary reasons for the choice are as follows: ①The Airbnb listings in the city are mainly concentrated within the main city of Nanjing, with a small number of suburbs; ②The central Nanjing is an area where important facilities gather. The built environment is relatively mature, and the influencing factors on the aggregation and distribution of Airbnb listings are more complex. The analysis of this area is helpful in exploring the distribution characteristics of Airbnb listings in Nanjing. The time range of the study is from July 2016 to October 2019.

### Variables and data collection

This Airbnb source data were derived from AirDNA, a third-party data platform, and were composed of the monthly data of Airbnb in Nanjing from 2016 to 2019, including the listing ID, type, location, price, income, booking status, and the host’s ID.

In current studies, factors related to housing distribution include urban centers [18, 24, 29], cultural attractions [11], and traffic accessibility [30, 31], service facilities [11], etc. Based on the characteristics of Nanjing, this study selected seven urban environmental factors, including subway stations, bus stops, cultural attractions, universities, shopping malls, living facilities, business apartments as independent variables for the study of the spatial distribution of Airbnb listings. The POI data of these independent variables were obtained from AMap, one of the largest mobile map and life service websites in China. The POI data types, acquisition methods, and quantities are shown in Table 1.

**Table 1:** Description table of selected variables.

Data	Description	Source	Number
Airbnb listings	Airbnb listing	AirDNA	12240
Cultural attractions	Tourist attractions, museums, memorials, artistic districts, historic districts	Amap POI	97
Subway Stations	Subway stations, Entrances and exits	Amap POI	320
Bus Stops	Bus Stops	Amap POI	1256
Shopping Malls	Shopping Malls	Amap POI	386
Living Facilities	Restaurants, cafes, fast food, market, grocery stores	Amap POI	24200
Universities	universities	Amap POI + Manual sorting	122
Business Apartments	Business Apartments	Amap POI	277

Traffic accessibility is a major factor affecting the distribution of Airbnb listings [32, 33]. Nanjing has the fourth-longest metro network in China. Therefore, subway travel has become a common choice for tourists. Nanjing is also famous for its well-developed bus transportation system, which is also a common mode of transportation for tourists.

Nanjing is an internationally well-known tourist city with rich cultural and historical resources. As an emerging way of travel and consumption, Airbnb may have a correlation between its spatial distribution and cultural attractions. Additionally, Nanjing is one of the cities of China that has the largest number of universities and is nationally known for the historical and cultural values and beautiful campuses, including Nanjing University, Southeast University, and Nanjing Normal University, which are also popular tourist attractions. Thus, Airbnb listing distribution may be related to the colleges and universities locations partly.

Because Airbnb guests are more willing to explore and experience local daily lives and communities, restaurants and life service facilities may also affect the spatial distribution of Airbnb listings. Since shopping spaces in city centers are often important destinations for visitors, the layout of large shopping malls may also have an impact on Airbnb’s distribution.

According to the preliminary analysis of Airbnb housing source data, the proportion of business apartment type housing source is always high. Therefore, business apartments are listed as an independent variable in this study. From the perspective of housing types, the main sources of Airbnb listings in Nanjing city are mainly business apartment, condominium, and service apartment, among which the proportion of business apartment is up to 50% (Table 2), which may have a great impact on the distribution of Airbnb.

**Table 2:** Statistical table of Airbnb listings in central Nanjing.

	2016		2017		2018		2019	
Business apartment	914	68.57%	2751	66.13%	3849	52.73%	6059	49.50%
Service apartment	9	0.68%	88	2.12%	679	9.30%	2059	16.82%
Condominium	294	22.06%	963	23.15%	2175	29.79%	3333	27.23%
Villa	30	2.25%	58	1.39%	67	0.92%	158	1.29%
Others	85	6.38%	296	7.12%	525	7.19%	632	5.16%
In total	1333	100.00%	4160	100.00%	7300	100.00%	12240	100.00%

**Fig 1:**
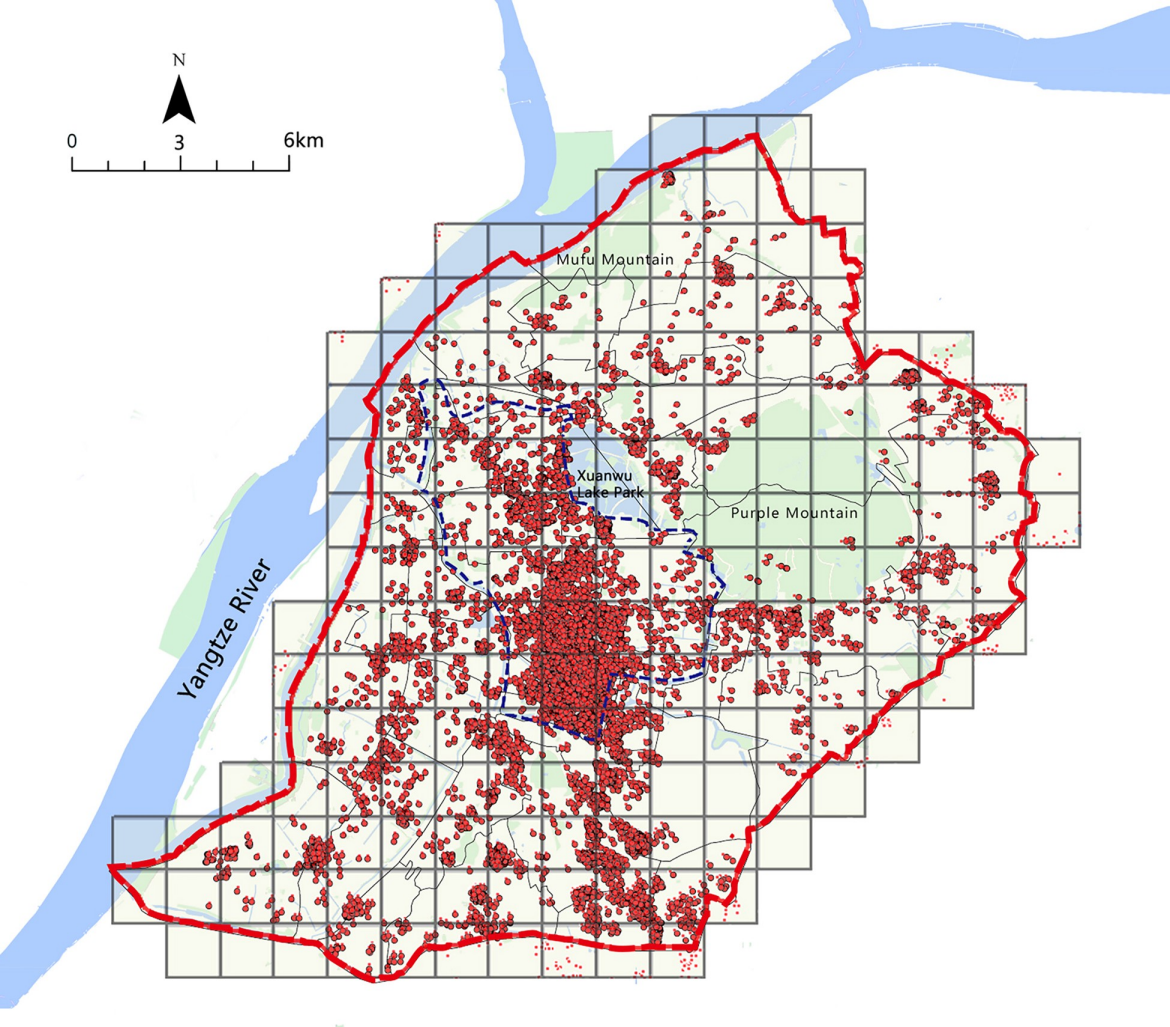
Distribution of Airbnb in central Nanjing. The central Nanjing was divided into 177 grids.

In order to quantify the spatial distribution characteristics of Airbnb more conveniently, this study divided the research scope (the central Nanjing) into 177 grids with a size of 1500m*1500m each. The number of Airbnb in each grid was defined as the dependent variable Y (Fig 1) and was calculated using Arcgis. And the distance from the center of the grid to the nearest subway station, cultural attractions, shopping malls, and universities were defined as independent variables X1-X4, while the number of bus stops, business apartments, and living facilities in each grid were defined as independent variables X5-X7 respectively. In addition, the urban construction area and non-construction area were distinguished through analyzing the satellite remote sensing images of the main city of Nanjing. In Arcgis, the percentage of construction area in each grid was calculated as the independent variable X8.

For count data such as the dependent variable in this study, negative binomial regression and Poisson regression are commonly used, in which the Poisson model is applicable to the case where the sample conforms to the Poisson distribution (i.e., expectation equals variance), while the negative binomial regression is applicable to the processing of data that is too discrete. In addition, when there are too many zero values in the sample data, zero-expansion Poisson regression or zero-expansion negative binomial regression should also be considered. In this study, ordinary Poisson regression, negative binomial regression, and zero-expansion negative binomial regression were used to process the data, respectively. After examining their results, zero-expansion negative binomial regression was selected as the most suitable model to analyze the data and obtained the variables with significant influence. Then, GIS was used to analyze the spatial relationship between the distribution of Airbnb housing source and urban built environment elements in order to discuss the coupling relationship between the location of Airbnb and urban structure.

## Results

### General distribution and agglomeration trends

**Fig 2:**
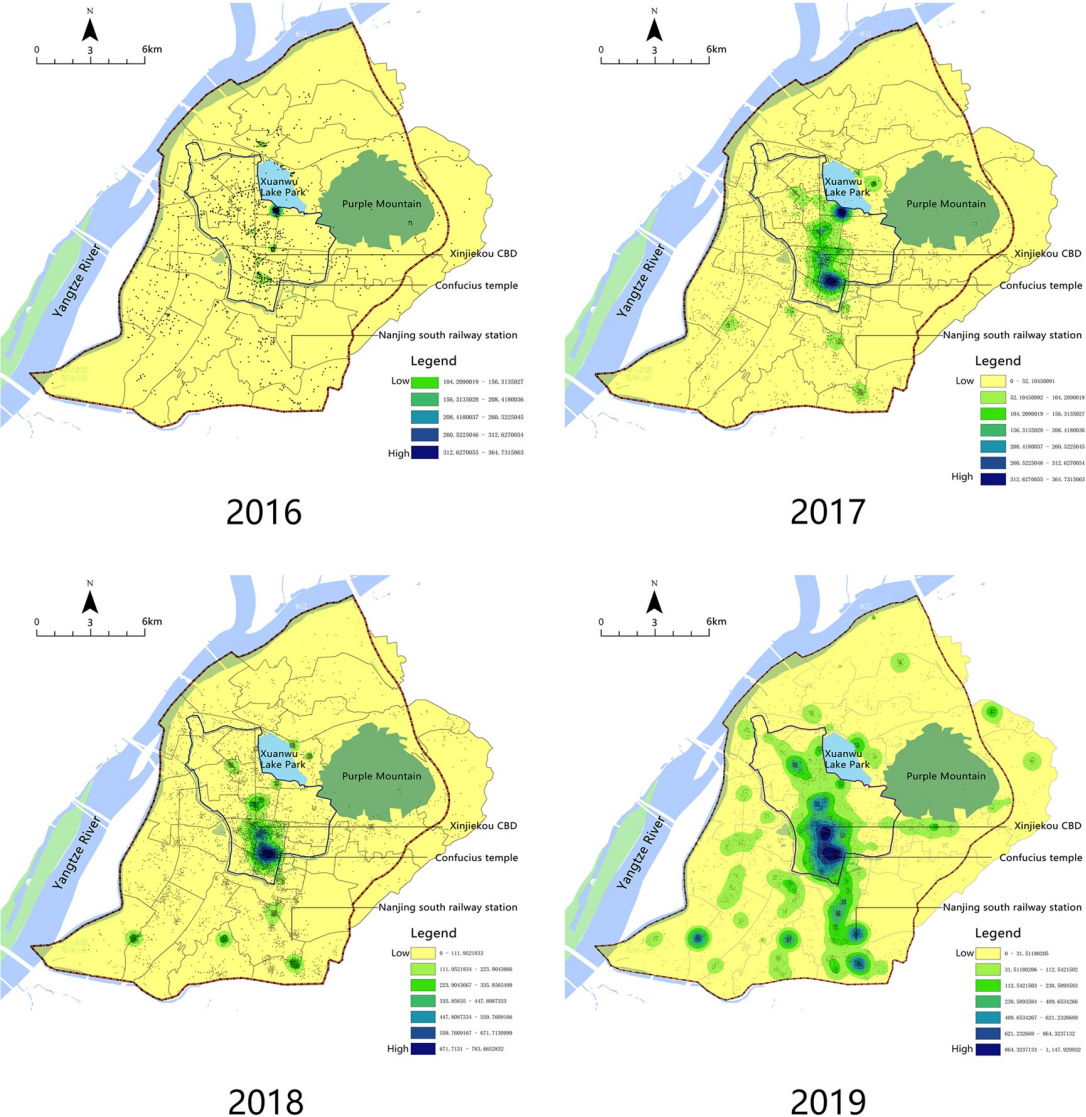
Kernel density map of Airbnb listings in central Nanjing 2016–2019.

Based on the change of Airbnb’s listing distribution from 2016 to 2019 (Fig 2), in 2016, the second year Airbnb entered China, the number of listing in central Nanjing is relatively small, and the locations are scattered, mainly distributed near the main scenic spots like Confucius Temple and Xuanwu Lake, without any cluster. Since 2017, housing sources have gradually gathered around Xuanwu Lake, Confucius Temple, and other important scenic spots, especially in the surrounding area of Confucius Temple. The Xinjiekou area has also begun to show the characteristics of agglomeration. In 2018, the number of listings increased rapidly, forming a central cluster in Xinjiekou—Confucius Temple area. By 2019, the distribution of Airbnb listings in the main urban area of Nanjing shows obviously clustering characteristics, including central clustering on the whole and "hallway-type" layout along with the rail transit on the periphery. In general, Airbnb listings in central Nanjing have grown very rapidly, and they have undergone a process of change from scattered locations to obvious clusters. From 2016 to 2019, in view of Airbnb listing spatial distribution, there is a certain regularity in its spread. The Airbnb space distribution in Nanjing can be summarized as four stages, namely the urban humanities scenic area, commercial center, transportation hub, and dispersive phase of development.

### Analysis of variables

Since the data of dependent variable Y (that is, the number of Airbnb in each grid) were a non-negative integer, the Poisson model was first considered for analysis. However, by statistical analysis of the dependent variable, it was found that its variance was 35066.28, far higher than its mean value of 70.75, which did not follow the assumption of Poisson model. Therefore, negative binomial regression was considered to be used for analysis. Results of negative binomial regression analysis conducted in Stata showed that the LR test results of alpha = 0 were: chibar2(01) = 9432.36, Prob> = chibar2 = 0.000, which indicated that the dependent variables in this study were overdispersed, not conforming to the assumption of Poisson distribution. Therefore, negative binomial regression should be used for analysis.

Because the dependent variable Y had a large number of zero values (27 out of 177 are zero values), negative binomial regression analysis with zero expansion was considered. Through observation of the grid distribution with the number of Airbnb was 0, it was found that most of them were located in areas with a large proportion of non-construction lands, such as forest land and water area. Therefore, the proportion of urban construction land in each grid was introduced as an expansion variable, and zero expansion negative binomial regression analysis was carried out in Stata. Vuong test showed that z = 1.71 Pr>z = 0.0440. Therefore, compared with ordinary negative binomial regression, it was more reasonable to select zero expansion negative binomial regression analysis.

Before regression analysis, the variance inflation factor test was performed on the independent variables. Variance inflation factor (VIF) of all the independent variables was less than 5 (Table 3), indicating that there was no multicollinearity, which confirmed the quality of the further analysis.

**Table 3:** VIF statistics of each variable.

	Variable	VIF
**x1**	Subway Station	1.57
**x2**	Cultural Attraction	1.27
**x3**	Shopping Mall	2.31
**x4**	University	1.65
**x5**	Bus Stop	2.56
**x6**	Business Apartment	2.48
**x7**	Living Facility	3.43
**x8**	Percentage of the construction area	2.51

Then, zero-inflation negative binomial regression was conducted in Stata. The expansion variable was x8 (the percentage of the construction area in the grid) (Table 4). The results show that the P values of seven among all eight variables, namely x1, x2, x3, x4, x5, x6, and x8, were all less than 0.1, which were statistically significant. The correlation coefficient of x1, x2, x3, and x4 was negative, indicating that they were negatively correlated with the number of Airbnb in the grid. In other words, the closer the center of the grid was to the subway station, shopping center, university and scenic spot, the greater the number of Airbnb is. The correlation coefficient of the number of x5 and x6 was positive, indicating that they were positively correlated with the number of Airbnb in the grid. This result suggests a larger number of Airbnb in places with more business apartments and bus stops.

**Table 4:** Regression results of zero-inflation negative binomial regression method.

	Variable	Coefficient	Standard Error	Z- Statistic	Probability
**x1**	Subway Station	-0.00040	0.000074	-5.34	0.000
**x2**	Cultural Attraction	-0.00028	0.000074	-3.69	0.000
**x3**	Shopping Mall	-0.00035	0.000178	-1.95	0.052
**x4**	University	-0.00016	0.000742	-2.11	0.035
**x5**	Bus Stop	0.04303	0.020555	2.09	0.036
**x6**	Business Apartment	0.15090	0.028772	5.24	0.000
**x7**	Living Facility	0.00046	0.000828	0.56	0.576
	**Inflate Variable**				
**x8**	Percentage of the construction area	-0.11816	0.045443	-2.60	0.009

#### Tourist attractions

First, Airbnb distribution is related to tourist attractions. This conclusion is similar to many studies. This research established a buffer zone of 1000m and 1500m for the main attractions in central Nanjing and counted the number of Airbnb listings. In the planar scenic spots like Xuanwu Lake and Purple Mountain, the edge created the planar buffer zone outward. In other small scenic spots, the circular buffer zone with the center of the scenic spot as the center of the circle was created. According to the statistics of the buffer zones, 9560 houses were distributed within the area of 1500m, accounting for 78%. 6,200 listings were in the buffer zone of 1000m, accounting for 51%. Among them, Confucius Temple, a historical and cultural scenic spot, had the strongest agglomeration effect on Airbnb, and together with Xinjiekou, the city center forms the core of Airbnb housing agglomeration.

#### Commercial and amenities

The rapid urban development in Nanjing since the 2000s results in the equalization in the spatial layout of community living facilities and almost all Airbnb locations were having convenient and adequate amenities, which is a possible reason that the correlation between the distribution of Airbnb listings and amenities did not pass the test. However, shopping malls in Nanjing are usually located in the main business centers of the city. The analysis results showed that the distribution of Airbnb houses was positively correlated with the layout of shopping centers, which might suggest that urban business center is an important factor affecting the distribution of Airbnb.

#### Transportation accessibility

**Fig 3:**
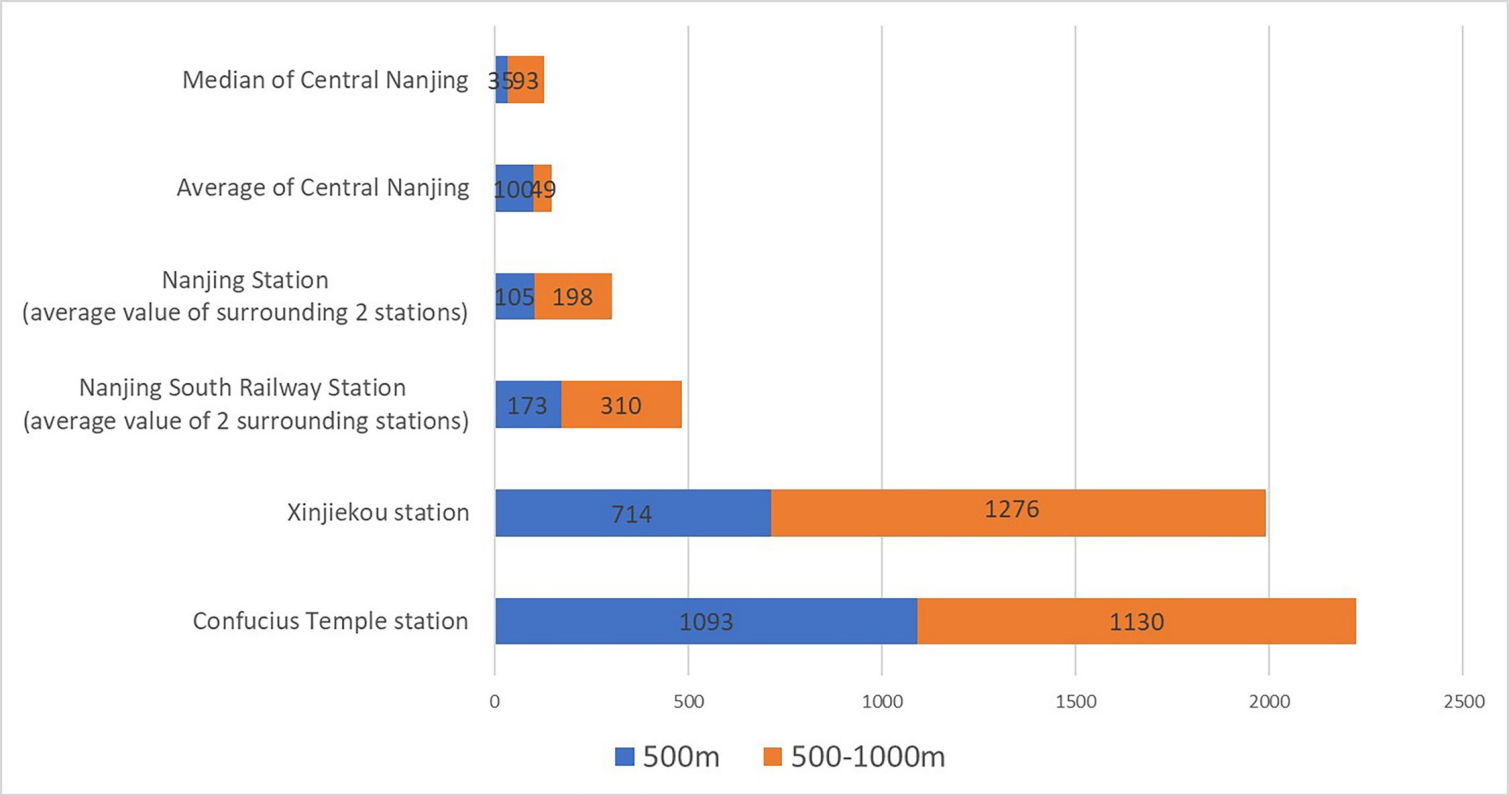
Distance analysis diagram of Airbnb distribution and subway stations.

The accessibility of urban rail transit is an important factor in explaining the clusters of Airbnb listings in Nanjing. According to statistics, the number of Airbnb listings within 500m from the subway station was 7321, accounting for 59.8% of the total in central Nanjing, while the number of listings within 1000m was 10,870, accounting for 80% (Fig 3). Therefore, the majority of short rent houses were clustered within walking distance of subway stations (1000m).

**Fig 4:**
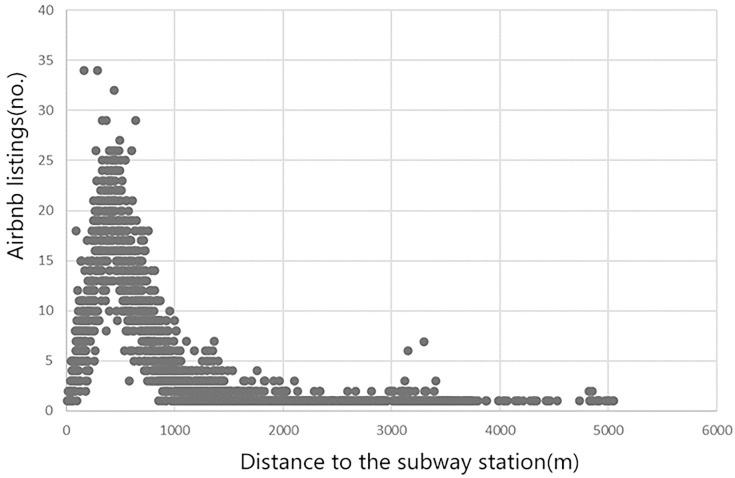
Relationship between Airbnb listing and the distance to the nearest subway station.

In order to understand the relationship between the number of Airbnb listings and the distance to subway stations, the nearest neighbor analysis tool in ArcGIS was used to find the distance between each listing point and the nearest subway station, and then aggregated them to generate scatter-distribution maps [12]. As shown in the scatter plot of the number and distance of Airbnb listings (Fig 4), there was an evident curve relationship between them, and the overall distribution was skewed, forming a U-shaped curve. In general, the closest distance between most Airbnb listings and subway stations was within 0-1000m. By analyzing this U-shaped curve, we found that listings within 500m of the subway station showed a linear upward trend, reaching a peak value around 338m, and then a linear downward trend. The prime cause is that most of the land around the subway station was used for other public functions. In most cases, a large number of commercial and residential buildings or residential buildings existed within the 300-500m range. Therefore, there was a significant upward trend within this range. After the maximum threshold of 338m, there was a downward curve because away from the subway station, the fewer the number of Airbnb was listed.

Most of the domestic and foreign tourists arrive in Nanjing from two railway stations, the Nanjing railway station, and the Nanjing south railway station. In this study, the buffer analysis of 500m and 1000m radius was done on the three nearest subway stations from Nanjing south railway station and Nanjing railway station, respectively. The results showed that the average of listings within 1000m to subway stations around Nanjing south railway station reached an average of 483, which was higher than the 303 at Nanjing railway station and higher than the average level of 149 at Nanjing urban subway stations. Accordingly, it suggests that “the convenience of rail transit to major high-speed rail stations” had a distinctly positive effect on the agglomeration of Airbnb listings.

#### Business apartments

The development speed of many large cities in China is still at a relatively high level, especially driven by the high-speed railway infrastructure and the developments of new areas. In city centers and new towns, a large number of business apartments and service apartments are often built, resulting in a high vacancy rate due to the lack of market demand. In China, the land use of business apartments and service apartments is commercial rather than residential, with a 40-year property right rather than 70 years. Therefore, they are more suitable to be transformed into Airbnb housing. With the gradual strengthening of policy management, residential Airbnb listings have been more restricted in the past two years, and professional hosts are more inclined to use business apartments.

**Fig 5:**
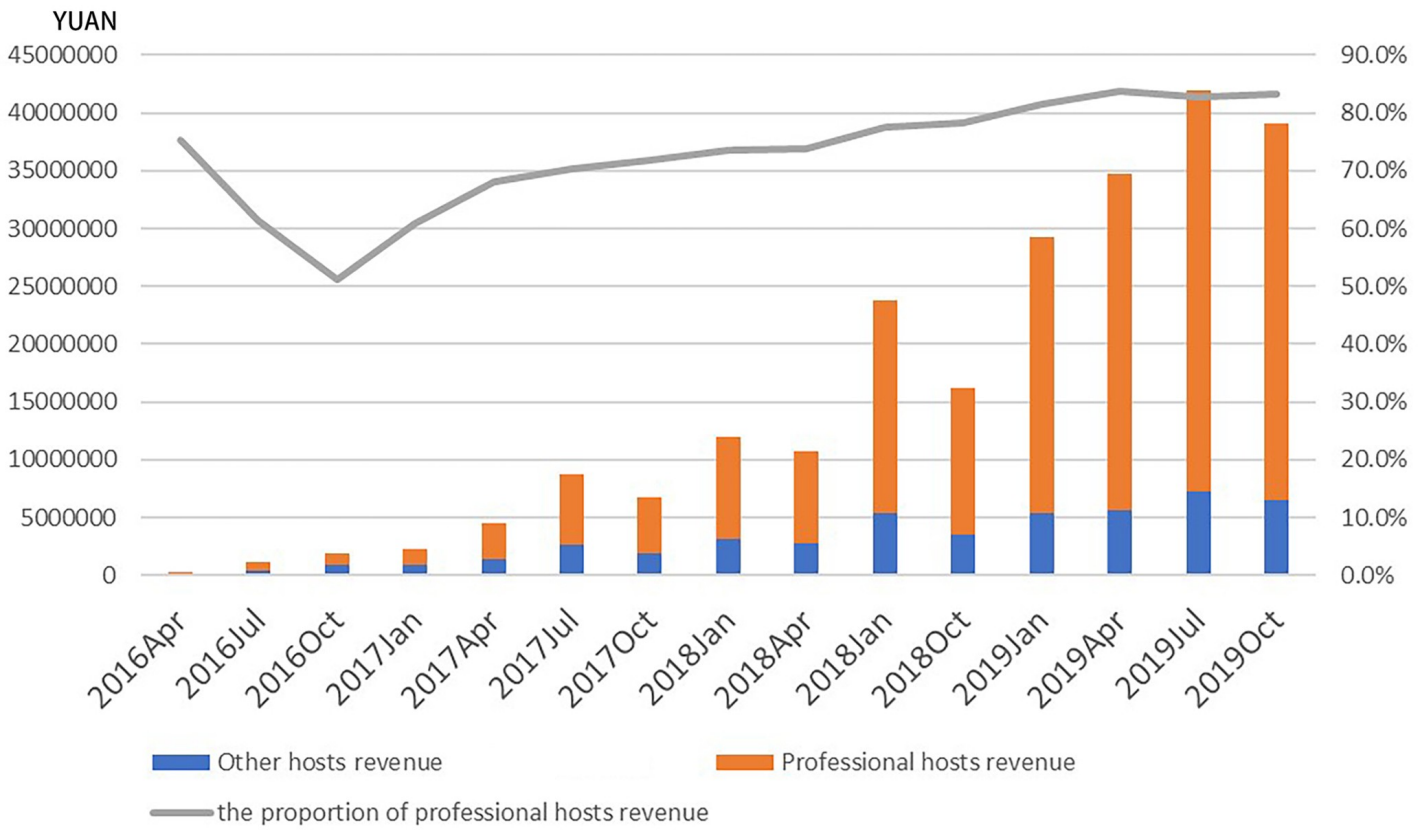
Changes in the income of Airbnb hosts in Nanjing.

The role of professional hosts is growing in Nanjing. Although the number of Nanjing vocational landlords accounted for 32% only, the number of housing that they held increased from 24% in April 2016 to 65% in October 2019. From the perspective of the host’s income (Fig 5), the phenomenon of professionalization and capitalization of Airbnb listings was more significant, with the proportion of professional host income reaching more than 80% at the highest in 2019. In terms of the change in the proportion of the housing income, the initial stage of the market (2016–2017) showed that the proportion of the professional host’s income decreased, but it maintained to rise gradually thereafter. All in all, the proportion of housing income of professional hosts shows the changing trend of a "V" shape, which suggested that the early market development allowed more landlords and entrepreneurs to participate in and make profits from it, reflecting a certain sharing nature. Nevertheless, with the stability of the market, professional hosts occupied most of the short-term rental market and exerted scale effect.

**Fig 6:**
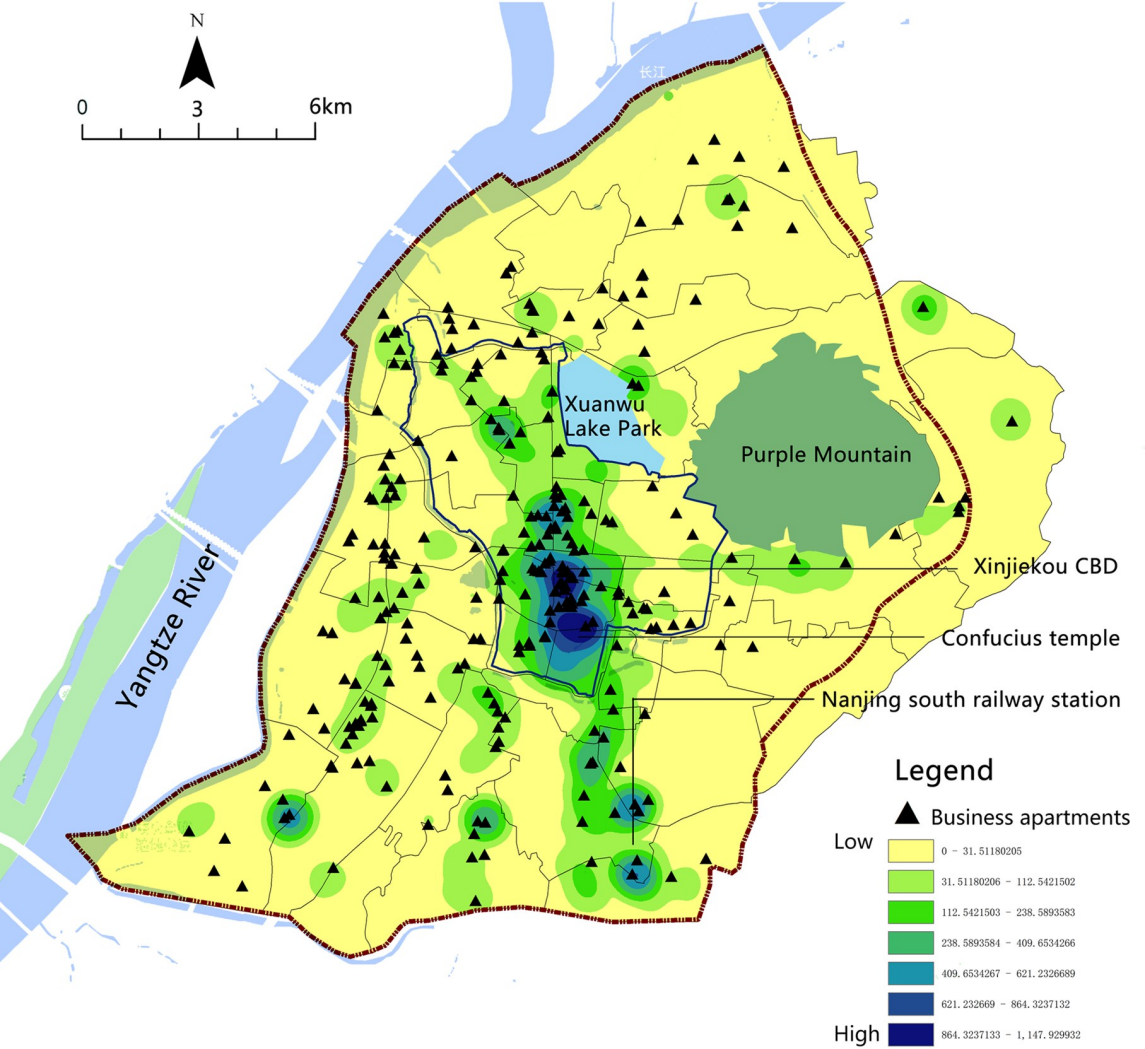
Comparison of the distribution of Airbnb listings and business apartments.

Meanwhile, more newly developed business apartments have been converted to Airbnb listings, which has become a new way of investing for some professional hosts. In particular, business apartments located in commercial centers, rail transit, and surrounding scenic spots naturally become ideal Airbnb listing choices (Fig 6).

## Discussion

The above regression results generally confirm that the spatial distribution of Airbnb has been closely related to factors including tourist attractions, the accessibility to urban rail transit, shopping malls, and business apartments. Meanwhile, all these factors are essential reflections of the way in which Nanjing has developed. To better understand the dynamic mechanisms behind the spatial distribution of Airbnb in Nanjing, this section further discusses that how the spatial distribution of Airbnb has been coupled with the urban spatial structure of Nanjing, the rent-gap, and the conversion of business apartments.

### The coupling between Airbnb distribution and urban spatial structure

**Fig 7:**
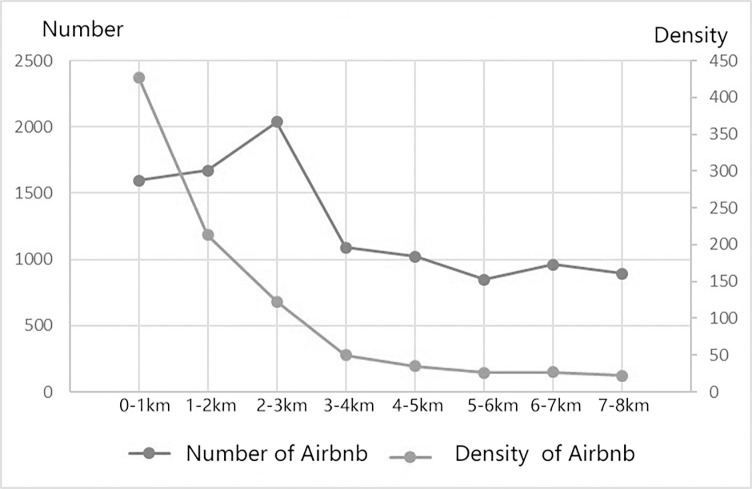
Relationship between Airbnb distribution and distance to the city center.

The distance to the city center has an important impact on the distribution of Airbnb listings. 50% of the listings in central Nanjing are concentrated in the old city. The southern part of the old city is the area with the highest listing density in Nanjing, and a cluster center is formed in the south of Xinjiekou. Besides Xinjiekou, the main scenic spots and high-speed railway stations are also the primary attraction for Airbnb in the city. It is shown volatility because of various factors that affect the spatial distribution of Airbnb. In addition, there is a negative correlation between the listing density and the distance from Xinjiekou (Fig 7).

**Fig 8:**
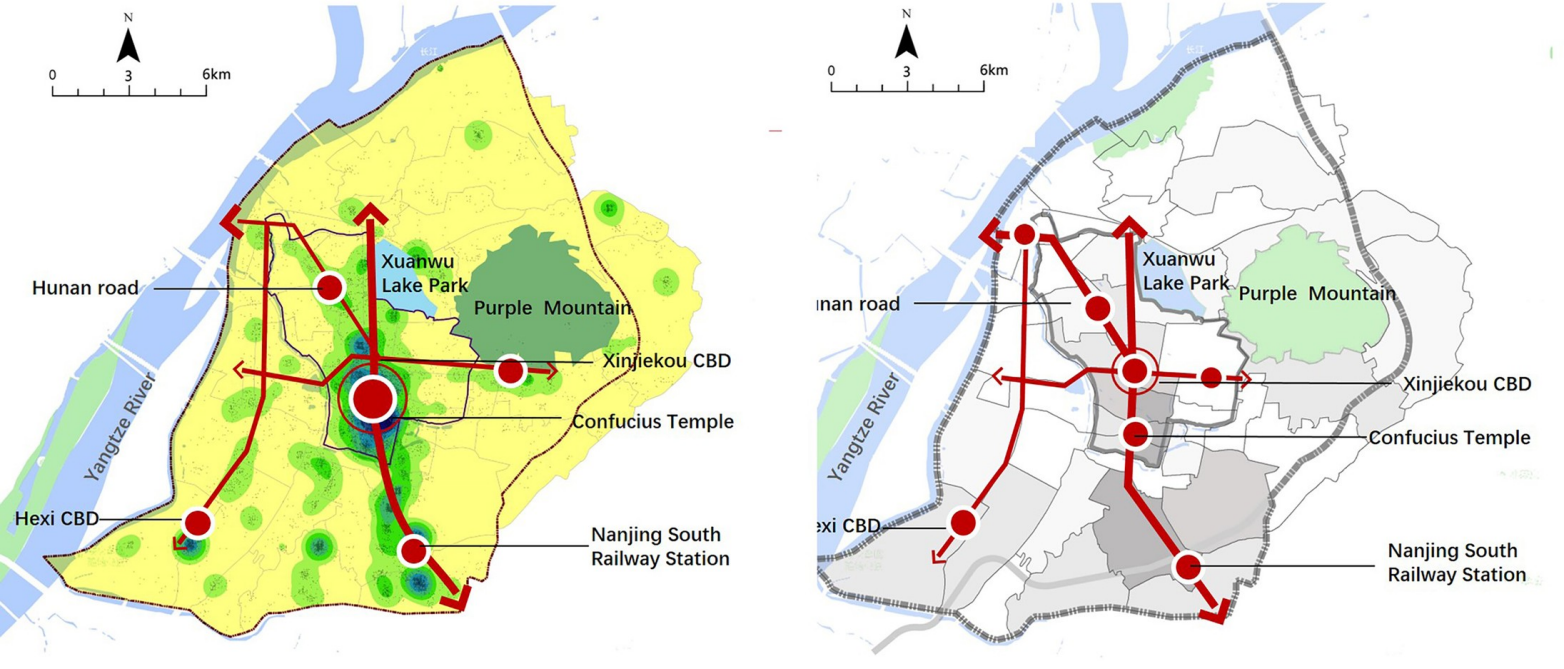
Comparison between the spatial structure of Airbnb (left) and that of central Nanjing (right).

The analysis shows that cultural attractions, shopping centers, and rail transit have significant positive effects on the agglomeration of Airbnb. The distribution of Airbnb housing resources, centering on the area of Confucius Temple and Xinjiekou, took the subway line as a corridor and formed cluster groups at subway stations, which generally showed the distribution characteristics of “central-corridor”. Airbnb distribution was formed spontaneously from the bottom up, showing a significant self-organization rule, and had a certain coupling relationship with the spatial structure of the city itself (Fig 8), which proved from one side that the urban spatial structure was the sequence of the interaction of various elements of the economy, society, and material environment.

### The correlation between the spatial distribution of Airbnb and rent-gap

**Fig 9:**
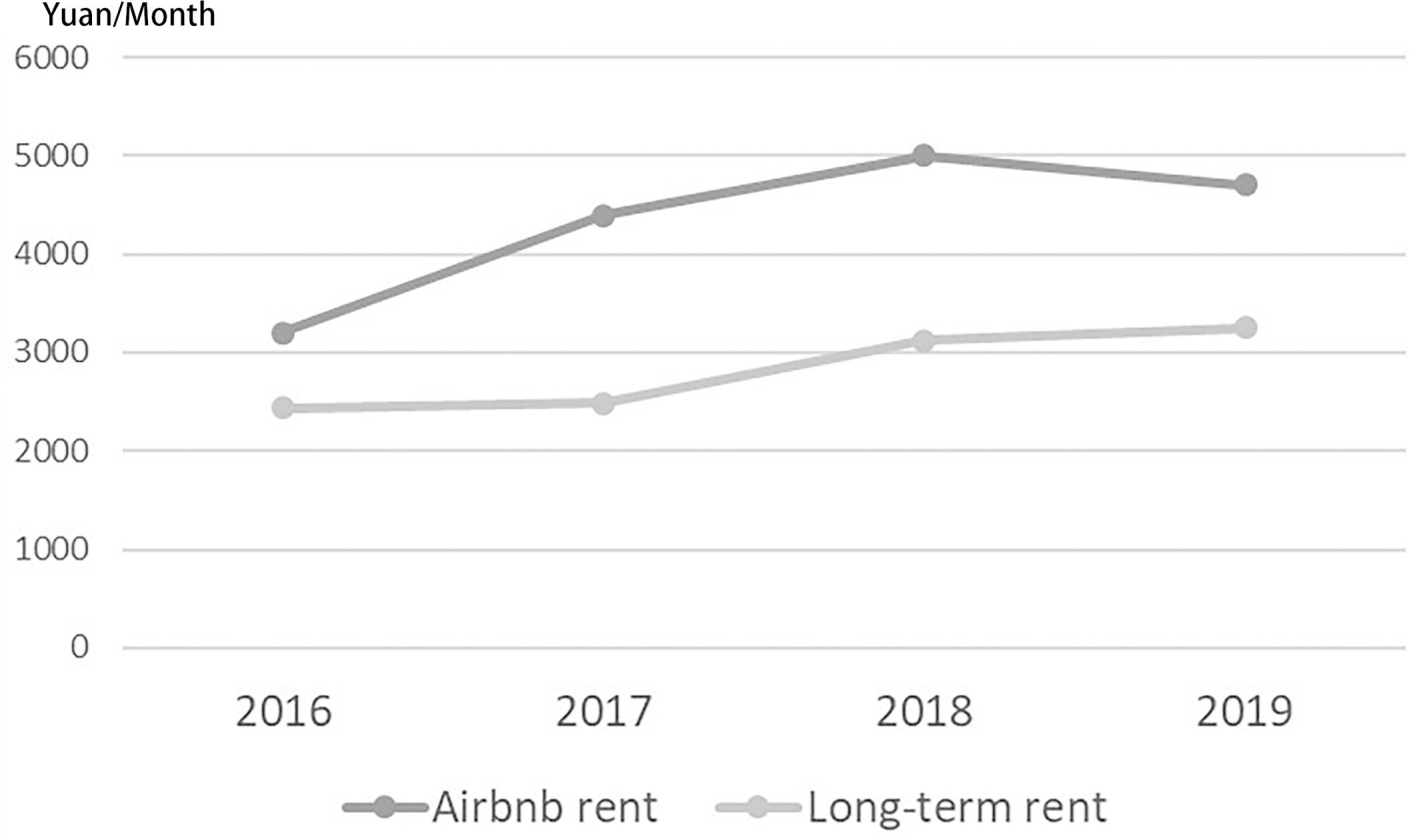
Rent gap between short- and long-term in Nanjing. Short-term rent data source: AirDNA, long-term rent data source: China Housing Market.

The “rent gap” is an important model to interpret the housing market from the perspective of economic drivers [21, 34, 35]. In the research field of Airbnb, researchers such as Wachsmuth and Weisler (2018) [4] and Yrigoy(2018) [8] also used the rent gap theory to explain the gentrification risks caused by Airbnb. As Airbnb converts numbers of long rents into short rents, a tendency of increasing commercialization of the landlord is presented, and the rentable housing eventually decreases and rents rise. For the hosts who focus on entrepreneurship, the monthly income of Airbnb listings may be much more than the income of traditional long-term rents, which encourages the hosts to convert the long-term rent into the short-term rent. The rent gap between the long rent and the short rent in Nanjing is shown in Fig 9. Initially, long-term rents of Nanjing urban residential buildings were in the slow increase. When the short-term rent platform created new potential land rent, the gap between potential rent and the actual rent increased. Fig 9 compares the gap between the median monthly income of active full-house listings in Nanjing (booked for at least one day in the month) and the average rent for long-term rentals. Overall, the rental income of Airbnb listings was significantly higher than that of long-term rentals, with annual revenue exceeding 150% of long-term rentals. It can be seen that Airbnb listings provided additional potential rent, and the rent gap encouraged landlords to use their houses as Airbnb listings. It constantly increased the number of listings, so as to obtain more revenue.

**Fig 10:**
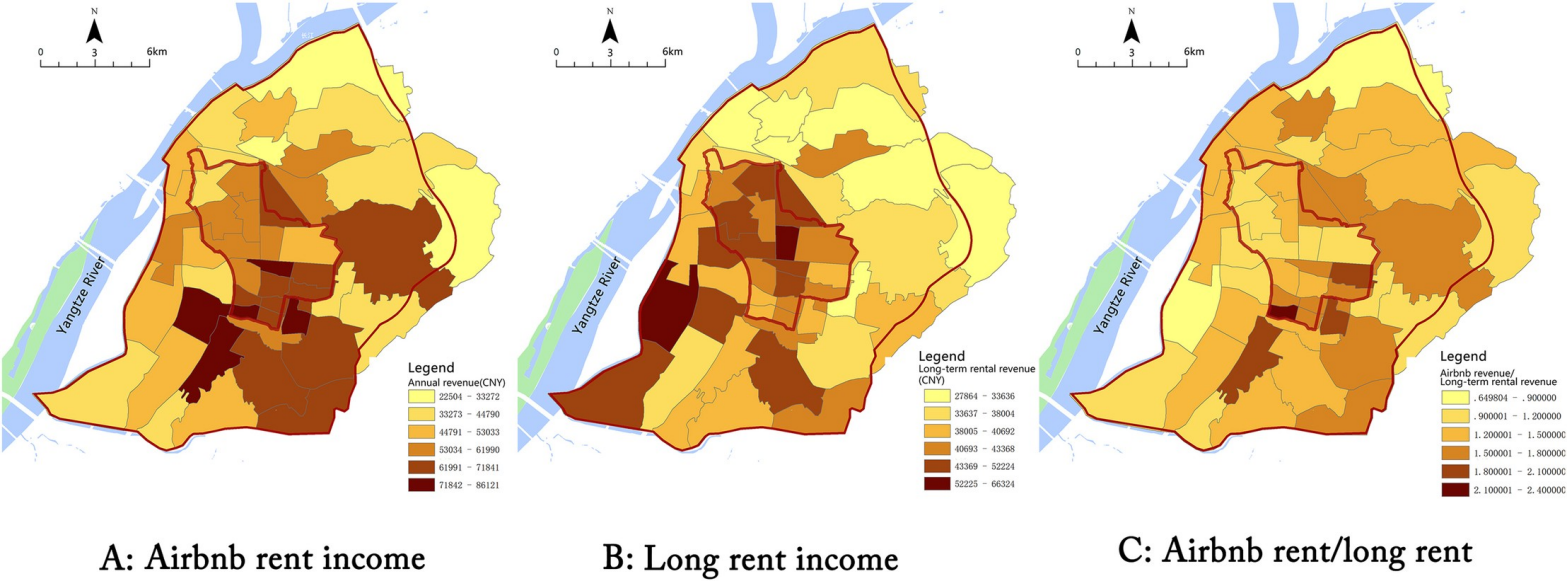
Spatial distribution of rent gap between Airbnb revenue and long-term rental revenue.

As shown in Fig 10, the spatial distribution of the rent-gap and the distribution of Airbnb are correlated. The high value of long-term rents was concentrated in the city center and sub-center, which was related to the city center system. However, the high value of Airbnb rents was concentrated in the tourist attractions and transport hubs, such as the Confucius Temple area, and Nanjing South Railway Station area, where Airbnb had a high density of distribution. The short-term rents have a stronger economic incentive effect on the conversion of long-term rents, and Airbnb rentals are more likely to continue to grow in this area in the future.

### The conversion of business apartments to Airbnb listings

In general, the excessive development of business apartments in cities further expands the Airbnb listings and becomes an important factor affecting the distribution of Airbnb. In the last decade, the main areas under construction in Nanjing were urban TOD areas and the sub-center area at the periphery of the old city, where a large number of business apartments had been built. Take the CBD of Nanjing South Railway Station as an example. In order to make full use of the land benefits around the high-speed railway station, plenty of commercial and residential mixed land and the commercial complex was planned around the Station. Since 2012, numerous service apartments have been developed and built on these mixed commercial and residential land. These projects had the spatial image of modern cities, reflecting the image of a gateway to the city’s high-speed railway station, and also became new hot spots for real estate investment in Nanjing. A large number of service apartments were used as Airbnb listings, especially the one-bedroom service apartment, which was suitable for operating Airbnb housing. Airbnb increased the possibility of space use and the blending of functions and improved the utilization efficiency of business apartments in the new city to a certain extent. However, this situation also makes Airbnb became more standardized and capitalized, losing the original intention of sharing and communication advocated by Airbnb.

## Conclusion

This study aims to solve the following questions: What are the characteristics of the spatial distribution of Airbnb listings in Nanjing city? What factors determine its distribution and expansion? We found that Airbnb listings were not randomly distributed in the city. There were significant and positive correlations between the spatial clustering of Airbnb and the built environments of cities. By comparing the spatial distribution of Airbnb and the spatial structure of central Nanjing, we found that there was a significant coupling between the two. There were a few reasons for it. First, the distribution of Airbnb was related to the distribution of scenic spots, rail transit stations, and shopping malls, while the layout of rail lines itself was related to the urban structure to some extent. Furthermore, the layout of business apartments was also related to the urban structure. Most of them were located in the city center, the new town center, and subway stations. Meanwhile, many of them were inside the large urban commercial complex and were part of the urban public center.

Similar to previous works [4, 5, 8], this study also found that the rent gap between long-term and short-term rent encouraged landlords to convert long-term rental housing sources into short-term rentals. Similarly, professional hosts in Nanjing also played important roles in the Airbnb market, with an increasing proportion of professional hosts, both in terms of quantity and income. Professional landlords had clear entrepreneurial minds. They preferred business apartments around commercial centers and main scenic spots. As the main promoter of Airbnb in Nanjing, professional hosts had important impacts on the spatial distribution and type of Airbnb. This study found that in Nanjing, a rapid-developing Chinese city, a large number of construction of business apartments provided great opportunities for the prosperity of Airbnb, which might be a unique spatial characteristic of Airbnb listings. In the future, there will be more Airbnb hosts choose business apartments as this type of housing, which will enhance the further commercialization and capitalization of Airbnb and boost its turn to boutique hotels.

Airbnb is an emerging urban-space-use mode with the Internet, as well as a model of sharing economy. The findings of this paper may provide positive references for urban renewal policies and public participation methods in China. The key issues are that the government and the industry should pay attention to how to normalize the short-term rental market like Airbnb and how to deal with the new emergence of urban space types under the Internet conditions.

There are many other factors affecting the distribution and development of Airbnb [36–38]. This paper studied the spatial distribution of Airbnb in Nanjing. Thus, the conclusion may be limited to the characteristics of Nanjing’s own development. For the rapidly developing Chinese cities, the conclusion of this paper needs to be further verified and expanded. In addition to further studying the differences in the distribution and types of short-term rentals in different types of Chinese cities, we should pay more attention to the rental shortage and gentrification of communities that Airbnb may cause, especially in the old urban areas of the metropolis in China.

## Supporting information

S1 Table Data for zero-inflation negative binomial regression. (XLSX)
